# The Regulation of Cellular Senescence in Cancer

**DOI:** 10.3390/biom15030448

**Published:** 2025-03-20

**Authors:** Xianhong Zhang, Yue Gao, Siyu Zhang, Yixiong Wang, Yitian Du, Shuailin Hao, Ting Ni

**Affiliations:** 1 State Key Laboratory of Reproductive Regulation and Breeding of Grassland Livestock, Institutes of Biomedical Sciences, School of Life Sciences, Inner Mongolia University, Hohhot 010070, China; zhangxianhong96@163.com (X.Z.); gyueyue1231@163.com (Y.G.); wyxwyx910@163.com (Y.W.); yitian_du@163.com (Y.D.); 2 Key Lab of Ministry of Education for Protection and Utilization of Special Biological Resources in Western China, School of Life Sciences, Ningxia University, Yinchuan 750021, China; zhangsiyu98@163.com

**Keywords:** cell senescence, caner, immunosenescence, senescence-associated secretory phenotype, senotherapeutics

## Abstract

Cellular senescence is a stable state of cell cycle arrest caused by telomere shortening or various stresses. After senescence, cells cease dividing and exhibit many age-related characteristics. Unlike the halted proliferation of senescence cells, cancer cells are considered to have unlimited growth potential. When cells display senescence-related features, such as telomere loss or stem cell failure, they can inhibit tumor development. Therefore, inducing cells to enter a senescence state can serve as a barrier to tumor cell development. However, many recent studies have found that sustained senescence of tumor cells or normal cells under certain circumstances can exert environment-dependent effects of tumor promotion and inhibition by producing various cytokines. In this review, we first introduce the causes and characteristics of induced cellular senescence, analyze the senescence process of immune cells and cancer cells, and then discuss the dual regulatory role of cell senescence on tumor growth and senescence-induced therapies targeting cancer cells. Finally, we discuss the role of senescence in tumor progression and treatment opportunities, and propose further studies on cellular senescence and cancer therapy.

## 1. Introduction

The number of cells in each system of the human body is in a dynamic balance. When some cells undergo normal senescence and apoptosis, new cells are produced to maintain the normal operation of tissues and organs. Various carcinogenic factors lead to excessive cell proliferation, disrupting the stable state of cell regulation and forming different cancer types [1]. These carcinogenic factors include not only intrinsic factors such as DNA mutations and epigenetic changes but also external factors such as carcinogens, viral microorganisms, and the tumor microenvironment (TME) [2]. Notably, the TME provides both the material and the energy basis for the growth of cancer cells, maintaining their unlimited proliferation, and creates conditions to evade immune clearance [3]. Recent studies have found that senescent cells exist in many stages of cancer within the TME [4]. This demonstrates that cancer cells can exhibit a senescence response, suggesting that inducing cancer cells to undergo the senescence process may be a viable method for treating cancer.

Cell senescence refers to irreversible cell cycle stagnation linked to cell morphology, secretory characteristics, and epigenetic changes [5]. Since 1961, Hayflick has found that even when normal human fibroblasts are given the most suitable conditions for growth, cell failure occurs when cell division reaches a certain algebra, and the cell cycle enters an irreversible stagnant state [6]. In line with Hayflick’s proposal, we currently understand that this phenomenon is due to telomere shortening and decreased telomerase activity as cells replicate, eventually reaching the “Hayflick limit,” and this senescence is known as replicative cell senescence. Senescent cells can survive but permanently stop growing and cannot re-enter the cell cycle. In 1997, Serrano et al. discovered that senescence is a tumor inhibition procedure that opened a new world for studying cell senescence in tumors [7]. Later studies found that senescence can be driven by excessive carcinogenic signal transduction or the loss of some tumor suppressor genes, namely oncogene-induced senescence [7]. Although oncogene-induced senescence (OIS) is an inherent tumor inhibitory mechanism, it can prevent primary precancerous cells from becoming fully transformed cancer cells [8]. However, senescent cells stably stagnate and produce complex secretory groups (called senescence-associated secretory phenotype (SASP)), which can promote tumor growth, recurrence, and metastasis [9]. A comprehensive analysis of the characteristics of senescence and cancer indicated a series of overlapping “characteristics.” For example, several characteristics of senescence (i.e., genomic instability, epigenetic changes, chronic inflammation, and metabolic disorders) are very similar to specific cancer characteristics, whereas other characteristics of senescence (i.e., telomere depletion and stem cell depletion) may inhibit cancer [10,11,12]. This indicates that cell senescence is closely linked to tumor occurrence and development. When cells undergo senescence, they can be used as an obstacle to tumorigenesis and play a role in tumor prevention or treatment. However, under certain conditions and backgrounds, malignant and non-malignant cells with persistent senescence can acquire tumor-promoting properties [13]. Consequently, cell senescence in cancer is a double-edged sword, and understanding the relationship between cancer cell growth and senescence is crucial for treating and preventing cancer.

## 2. The Causes of Normal Cellular Senescence

**Figure 1:**
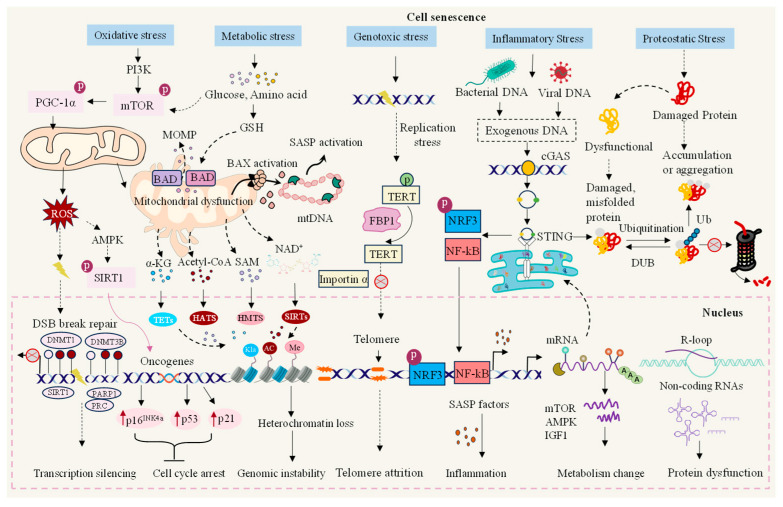
Endogenous and exogenous factors of cell senescence. Various endogenous and exogenous stimuli can regulate intracellular gene expression and metabolism through a series of signaling pathways, interfere with epigenetic processes, and cause genome stability and protein biological function abnormalities, thus inducing cell senescence. PI3K: posphoinositide 3-kinase; PGC-1α: peroxisome proliferator-activated receptor gamma coactivator 1-α; mTOR: mechanistic target of rapamycin; MOMP: mitochondrial outer membrane permeabilization; GSH: glutathione; SASP: senescence-associated secretory phenotype; BAD: Bcl-xL/Bcl2-associated death promoter; BAX: Bcl2-associated X; mtDNA: mitochondrial DNA; ROS: reactive oxygen species; AMPK: adenosine monophosphate-activated protein kinase; SIRT1: sirtuins 1; α-KG: alpha-ketoglutarate; SAM: S-adenosylmethionine; NAD+: nicotinamide adenine dinucleotide; DSB: double-strand break; DNMT1: DNA methyltransferase 1; DNMT3B: DNA methyltransferase 3 beta; PARP1: poly (ADP-ribose) polymerase 1; PRC1: protein regulator of cytokinesis 1; TETs: thymic epithelial tumors; HATS: heteromeric amino acid transporters; HMTs: histone methyltransferases; SIRTs: sirtuins; Kla: lysine lactylation; AC: adenylyl cyclase; Me: methylation; TERT: telomerase reverse transcriptase; FBP1: fructose 1,6-bisphosphatase 1; NRF3: nuclear factor erythroid 2-related factor 3; NF-κB: nuclear factor κB; cGAS: cyclic GMP-AMP synthase; STING: stimulator of interferon genes; IGF1: insulin-like growth factor 1; Ub: ubiquitin; DUB: deubiquitylating enzyme.

Hayflick reported that even when normal human fibroblasts are provided with optimal growth conditions, cell failure occurs when cell division reaches a certain threshold, and the cell cycle enters an irreversible stagnant state [6]. According to Hayflick’s findings, we currently comprehend that this phenomenon is because as cells replicate, telomere shortening and telomerase activity decrease, eventually causing cells to reach their “Hayflick limit,” which is a form of replicative cell senescence. Subsequently, further research has revealed that, in addition to senescence caused by telomere shortening, cells are subjected to various internal and external pressures during growth. These include carcinogenic activation, radiation or chemotherapeutic drugs, and oxidative and genotoxic stress, which can lead to DNA or mitochondrial dysfunction [14]. In some cases, programmed cell death may occur if the damage cannot be repaired. Alternatively, these cells can steadily exit the cell cycle and become senescent [15]. This means that cell senescence can be highly variable and heterogeneous, and cells can experience four main types of senescence depending on the inducer: replicative senescence (RS), oncogene-induced senescence (OIS), stress-induced premature senescence (SIPS), and therapy-induced senescence (TIS) [16,17,18]. Cell senescence causes growth arrest, chromatin remodeling, DNA damage response (DDR), specific morphological changes, and metabolic alterations [19]. Senescent cells are accompanied by several significant features (Figure 1), including increased expression of the cyclin-dependent kinase inhibitor p16 or p21, elevated activity of β-galactosidase, and increased secretion of pro-inflammatory cytokines, chemokines, growth factors, and proteases as part of the senescence-associated secretory phenotype (known as SASP) [20].

### 2.1. Telomere Shortening

Telomeres are located at the ends of chromosomes and are generally composed of repeating tandem sequences (TTAGGG). In the absence of a telomere maintenance mechanism (such as telomerase expression or telomere recombination), normal DNA replication machinery cannot completely replicate the DNA ends of chromosomes; as a result, cells lose approximately 50–100 DNA base pairs every time they undergo replication. The binding of telomeres to protein complexes helps form a lasso-like structure, which regulates the biological function of telomeric DNA. Activation of the telomere DDR leads to the formation of telomere-associated foci (TAF) or telomere dysfunction-induced foci (TIF). The prevailing hypothesis is that it is not telomere dysfunction per se that leads to senescence, but rather the telomeric DNA damage response (TDDR) activated by telomere dysfunction that drives cell senescence [21]. Consequently, telomere length is regarded as an indicator of cellular senescence. Generally, telomere lengthening is facilitated by telomerase reverse transcriptase (TERT), which uses RNA as a template to synthesize and extend telomere DNA. It has been discovered that fructose-1,6-bisphosphatase (FBP) dephosphorylates the serine 227 (S227) site of TERT through interaction with its hydrophobic residue N273, thereby inhibiting the nuclear translocation of TERT, significantly reducing telomerase activity, shortening telomere length, and promoting cell senescence [22]. Indeed, telomere length serves as a key regulator of senescence, influencing the expression of a wide range of genes. Senescence mediates the expression of nearly a thousand genes through telomere length [23]. Telomerases can extend telomere length. Recent research has shown that T cells can acquire telomeres from extracellular vesicles secreted by antigen-presenting cells (APCs), leading to the extension of their telomeres. T cells that acquire telomeres become long-lived central memory cells, akin to stem cells, whereas the remaining T cells undergo the senescence process [24]. This highlights the critical role of telomeres in regulating the senescence process.

### 2.2. Genomic Instability and Epigenetic Change

The integrity and stability of the genome are threatened by both exogenous agents (such as chemical, physical, and biological factors) and endogenous challenges (such as DNA replication errors, chromosome segregation defects, and oxidative damage). These threats can lead to damage to both nuclear and mitochondrial DNA [11]. When DNA damage or genomic instability occurs, cells attempt repair; however, when damage is persistent and exceeds a certain threshold, repair becomes difficult, potentially leading to biological outcomes such as senescence and cancer. Nuclear DNA damage is a primary cause of cell senescence, potentially leading to DNA accumulation in extra-nuclear structures and the formation of micronuclei (MN), which are indicators of genomic instability and signs of cell senescence and disease [25]. In addition to nuclear DNA damage, mitochondrial DNA (mtDNA) is particularly susceptible to damage or mutation due to the lack of a sophisticated repair system and its proximity to the main source of reactive oxygen species (ROS). Researchers used nanopore Cas9-targeted sequencing (nCATS) to map and quantify mtDNA deletion mutations, finding that many mtDNA deletions in the human substantia nigra increase with age [26]. This indicates that deletion mutations of mtDNA can serve as an indicator of tissue senescence. Notably, a recent study found that an apoptosis-related increase in mitochondrial outer membrane permeabilization (MOMP) occurs in mitochondria without inducing apoptosis, a key feature of cell senescence referred to as minority MOMP (miMOMP). During senescence, miMOMP stimulates SASP by activating the cGAS-STING pathway [27]. In animal models and organs, the accumulation of mtDNA mutations can lead to the depletion of nicotinamide adenine dinucleotide (NADH)/nicotinamide adenine dinucleotide (NAD+) redox state during intestinal senescence, thereby impairing Wnt/β-catenin signaling and depleting intestinal stem cells to induce senescence [28]. These studies suggest that genomic instability, mediated by the DNA damage response, regulates cell senescence through a series of signaling cascades.

Epigenetic changes such as alterations in DNA methylation patterns, post-translational modifications of histones, chromatin remodeling, and non-coding RNA (ncRNA) function contribute to senescence. Recent research has shown that DNA damage influences senescence at physiological, cognitive, and molecular levels, such as epigenetic landscape erosion, cell differentiation, advancement of the DNA methylation clock, and accelerated mammalian senescence due to loss of epigenetic information [29]. Similarly, resetting the epigenetic information has shown that cellular responses to double-stranded DNA breaks induce epigenetic changes that accelerate the production of senescence-related markers, though epigenetic remodeling can reverse these changes [30]. This indicates that abnormal epigenetic changes may disrupt cellular senescence.

As the transcription process directly regulates protein levels in cells, ensuring the correct synthesis of proteins is crucial for cell senescence. Eukaryotic precursor mRNA must undergo complex processing (5′ end capping, intron and exon splicing, and 3′ end processing) to mature and perform its biological functions. Mature mRNA includes the coding region, untranslated region (UTR), poly (A) tail, and 5′ cap structure. Among these, the non-coding regions of mRNA (such as the intron and 3′ untranslated region) have been discovered to play roles in cell senescence. Important RNA-binding proteins can regulate cell senescence by altering the mRNA untranslated regions. For example, our previous study found that the retention level of over 800 introns was continuously upregulated or downregulated in senescent cells, where splicing factor U2 small nuclear RNA auxiliary factor 1 (U2AF1) combined with the last intron of *CPNE1* to promote effective splicing in young cells. U2AF1 downregulation increases the retention of the last intron of the downstream target gene *CPNE1*, resulting in fewer spliced transcripts, reduced RNA degradation, and decreased copine1 (CPNE1) protein production in the nucleus, leading to senescence-related phenotypes [31]. Additionally, more genes tend to use the distal poly (A) (pA) site than the proximal ones in several cell senescence modes, resulting in the overall elongation of the 3′ UTR and decreased gene expression. Among them, the mRNA of the RRAS2 gene (a member of the Ras superfamily involved in multiple signal transduction pathways) more likely to utilize use the long 3′ UTR reduces the level of RRAS2 protein and leads to senescence [32]. These studies reveal dynamic changes in the non-coding regions of mRNA in regulating cell senescence.

### 2.3. Metabolic Changes

Cell senescence can be influenced by metabolic processes such as mitochondrial dysfunction, NAD(+) depletion, and hyperglycemia. Additionally, cell senescence can regulate phenotypes related to metabolic functions. Glucose is a crucial energy sources for cells to maintain their viability. Many studies have shown that cells cultured at high glucose levels exhibit accelerated senescence. For instance, high glucose levels lower SIRT3 expression and promote fibroblast senescence. Conversely, SIRT3 overexpression prevents senescence induced by high glucose [33]. This suggests that limiting the amount of glucose consumed by cells can delay senescence. In a recent study, pyruvate dehydrogenase kinase 4 (PDK4), a key enzyme in the metabolic reprogramming of senescent cells, was identified. Senescent cells exhibited increased PDK4-dependent aerobic glycolysis and lactic acid production. PDK4 inhibition reduces the severity of DNA damage and inhibits the SASP. Similarly, in mice, PDK4 inhibition alleviated senescence-induced dysfunction and prevented weakness later in life [34].

In addition to glucose metabolism, serine metabolism regulates endothelial cell senescence. Researchers found that PHGDH—a key protein that regulates serine biosynthesis—inhibits the acetylation and autophagy degradation of pyruvate kinase M2 (PKM2) at lysine site 305 (K305) catalyzed by acetyltransferase PCAF through interaction with PKM2. In contrast, PHGDH promotes the acetylation of lysine (K433) of PKM2 catalyzed by p300, stimulates the phosphorylation of histone H3 at threonine 11 (H3T11), and further regulates the transcription of senescence-related genes. Exogenous serine supplementation in endothelial cells with decreased PHGDH expression delays senescence [35]. Additionally, tumor cells and regulatory T cells (Tregs) can influence T-cell senescence and tumor immune function by regulating lipid metabolism imbalances in conventional T cells. Specifically, the MAPK and STAT1/3 signaling pathways synergistically promote the expression of cytosolic phospholipase A2 alpha (cPLA2α) in T cells, leading to changes in lipid metabolism, lipid droplet (LD) accumulation, and senescence phenotype [36]. These studies have disclosed that intracellular nutrients and various metabolic pathways interfere with cell senescence development.

### 2.4. Abnormalities in Signal Transduction

#### 2.4.1. AMPK/mTORC Signaling Pathway

AMPK is a critical protein that maintains metabolic homeostasis in the body. Senescence impairs AMPK regulation, leading to reduced expression of the regulatory subunit PRKAG1 (γ1). In contrast, aged animals with increased AMPKγ1 expression due to transgenic technology exhibit feeding and fasting responses similar to those of young adults, which effectively improves metabolic health and lifespan. This suggests that selective stimulation of the AMPKγ1 complex can prevent senescence [37]. Similarly, inhibition of poly (ADP-ribose) polymerase-1 (PARP1) in aged Drosophila prolonged lifespan. Knockdown of PARP1 in skeletal muscle increased AMPKα activity by down-regulating the poly (ADP-ribosylation) modification of AMPK. This up-regulation of PGC-1α and PTEN-induced putative kinase 1 (PINK1) resulted in accelerated mitochondrial turnover, increased muscle metabolism, and enhanced function, elucidating the involvement of PARP1/AMPK in senescence regulation [38]. These studies suggest that reduced AMPK activity may accelerate cellular senescence, while enhanced AMPK activity through drugs, transgenics, dietary modification, or exercise appears to delay senescence.

Much evidence has shown that mitochondrial dysfunction, loss of protein homeostasis, genomic instability, and telomere shortening lead to imbalanced mTORC1 signaling and induce cellular senescence [39]. Indeed, early studies found that lifespan could be extended in yeast, worms, and drosophila by inhibiting mTORC1 or mTORC1 downstream signaling pathways, including S6K and translation initiation factors [40,41]. Moreover, deletion of the mTORC1 subunit (S6K1) or downstream substrates prolonged the lifespan of mice in both normal mice and genetic mouse models [42,43]. These studies all suggested that rapamycin, a potent chemical inhibitor of mTORC1, might act to extend lifespan. This was the case, as rapamycin treatment extended the lifespan of model organisms, including yeast, worms, flies, and mice [44,45]. A recent study found that overexpression of arginine-rich dipeptide repeat (DPR) leads to nucleolar stress (NS), mTOR overactivation, and r-protein accumulation, which in turn accelerates senescence and triggers premature death in mice [46]. Similarly, studies in mouse models have found that increasing trophic signaling in cells leads to parenchymal damage, inflammation, and shortened lifespan. Increased mTOR activity promoted the expression of inflammatory molecules in mice, which accelerated senescence and shortened their lifespan by about 20%. Moreover, blocking the immune response that causes inflammation ameliorates the symptoms of senescence. Interestingly, this process also applies to humans [47]. These studies suggest that hyperactivation of mTOR signaling due to endogenous and exogenous signaling stimuli leads to imbalances in intracellular oxidative stress, inflammation, and protein homeostasis and further accelerates cellular senescence.

#### 2.4.2. Insulin-like Signaling Pathways

The insulin/IGF-1 signaling pathway (IIS) was the first signaling pathway discovered and widely validated for its role in regulating senescence [48]. Inhibition of IIS has been shown in several organisms to improve mitochondrial function and enhance cellular adaptation to oxidative stress, ultimately mitigating the senescence process and prolonging longevity. DAF-2 is the homolog of the insulin-like growth factor 1 (IGF-1R) receptor in *Caenorhabditis elegans*. As early as 1993, researchers found that mutations in *daf-2* in *Caenorhabditis elegans* were involved in the transition from normal developmental progression to the dauer stage, dramatically extending the adult lifespan [49]. Recent studies provide more direct evidence for the involvement of IIS in organismal senescence. One study used RNA sequencing to analyze 41 mammalian species, including the long-lived naked mole rat, Bryde’s mouse-eared bat, and bowhead whale, to characterize their longevity gene expression profiles. A quantitative meta-analysis of 92 publicly available datasets revealed shared mechanisms of longevity across species, including down-regulation of IGF1 expression and up-regulation of mitochondrial translational genes, as well as unique traits such as the innate immune response and cellular respiration [50]. This suggests that down-regulation of IGF1 expression may be sufficient to delay senescence. Intracellular IIS activity is initiated by two receptor tyrosine kinases, the insulin receptor (IR) and the IGF1 receptor, which, upon ligand binding, phosphorylate the IRS protein, a key downstream mediator of the pathway. Another study found that neuron-specific IRS1 deletion in knockout mice mediated changes in the integrated stress response in mitochondria. This was demonstrated through various metrics such as lifespan, glucose tolerance, islet tolerance, body weight, and energy expenditure, and was effective in improving the health of aged male mice [51]. This suggests that abnormal IIS signaling is closely related to cellular senescence and that regulating key signaling molecules of the IIS may offer potential interventions for both organismal and cellular senescence.

#### 2.4.3. Sirtuin-Mediated Signaling Pathway

SIRTs are class III histone deacetylases dependent on nicotinamide adenine dinucleotide, found in many organisms, and regulate crucial biological processes like genome stability maintenance, metabolism regulation, and stem cell modulation [52]. SIRT1, SIRT2, SIRT3, SIRT6, and SIRT7 are true protein deacetylases, while SIRT4 and SIRT5, which do not deacetylate, remove other acyl groups from lysine residues. The widespread localization of sirtuins suggests that the SIRT family regulates various cellular functions. SIRT2 plays a crucial role in life extension under yeast dietary restriction [53]. Recent transcriptomic and proteomic analyses of senescent crab-eating monkeys’ hearts showed that SIRT2 delays myocardial senescence by deacetylating STAT3, suggesting that SIRT2 gene therapy could reverse cardiac senescence [54]. Exosomes from mesenchymal stem cells can delay brain senescence by increasing SIRT1 expression [55]. These studies suggest that different sirtuin members regulate cellular senescence in various ways. To explore how sirtuins regulate senescence, researchers used genome-targeted editing to knock out SIRT1-7 in human embryonic stem cells and differentiated them into mesenchymal stem cells. Deletion of any SIRT1-7 member in MSCs caused accelerated senescence. Further investigation showed that deleting any sirtuin increased histone acetylation and amplified activated enhancer states in chromatin regions sensitive to sirtuin deletion [56]. This indicates that various sirtuin family members are involved in nuclear epigenetic regulation. Evidence shows that NAD+ levels and sirtuin activity decrease with age and in senescent or high-fat-diet animals [57]. Fasting and exercise, however, increase NAD(+) levels [58,59]. Combining NAD(+) precursor supplementation with inhibition of the tryptophan kynurenine (K) pathway significantly improves senescence, promotes healthy aging, and extends lifespan [60]. Overall, these studies suggest that sirtuins and NAD(+)-mediated epigenetic modifications or signaling influence the senescence process.

## 3. Senescence of Immune Cells

In 1969, Warford proposed the “immunological theory of senescence,” which revealed that a decline in immune system function was a significant factor contributing to body senescence [61]. Senescent cells include immune cells such as T cells, macrophages, and natural killer cells, and they can also induce further senescence in other immune cells, thereby accelerating the aging process [62]. As individuals age, there is an accompanying decline in immune organ function, immune cells, and antigen response, which results in decreased immune surveillance and is associated with many age-related diseases [63]. Recent research provides direct evidence that immune cell senescence impairs immune function and promotes damage to other immune organs and tissues in mice. Transplanting senescent immune cells can further promote senescence and tissue damage. Conversely, transplanting “young” immune cells can partially reverse senescence [64]. This study suggests that immune cell senescence has significant potential to drive organismal senescence and increase the risk of age-related diseases.

### 3.1. Innate Immunity in Senescence

The body’s immune system comprises the innate and adaptive systems. The first line of defense primarily consists of monocytes/macrophages and natural killer (NK) cells. Researchers have analyzed the single-cell transcriptome and T-cell receptor (TCR) transcriptome of human peripheral immune cells from newborns to the frail elderly. It was found that genetic heterogeneity of immune cells increases with age and senescence, with immune cells undergoing senescence at varying rates and patterns [65]. This suggests that the body’s senescence process is accompanied by immunosenescence driven by differential gene expression in immune cells, including monocytes. Senescence impacts the diversity and functional properties of NK cell subsets in both mice and humans [66]. For instance, during senescence, perforin release and binding to target cells at immune synapses are significantly reduced, diminishing NK cell cytolytic activity [67]. An 11-year study of Japanese middle-aged and older adults found that immune regulatory cells increased with age but failed to maintain immune balance and even inhibited NK cell function [68]. This suggests that age-related changes may account for the reduced cytotoxicity of NK cells in the elderly. DCs originate from hematopoietic stem cells in the bone marrow, circulate in the blood, and reside in tissues where potential antigens are encountered [69]. Studies have identified key features of DC aging, including decreased migration rate, reduced antigen uptake, and diminished T-cell antigen processing [70,71]. In a study of peripheral blood DC subsets in healthy adults, it was found that the pagocytic function of DCs declines with age [72]. In summary, organismal senescence is accompanied by the senescence of the innate immune system, which exacerbates overall senescence and increases disease risk.

### 3.2. Adaptive Immunity in Senescence

The adaptive immune system consists of T and B lymphocytes and includes both cellular and humoral immunity. T cells are particularly susceptible to senescence. As the body ages, thymic degradation impairs the proliferation of naïve T cells, leading to a reduction in naïve T-cell populations and an increase in memory T-cell pools, thereby decreasing the available naïve T-cell repertoire [73]. Immunosenescence is associated with TCR and coreceptor dysfunction, with the loss of the CD28 receptor being a hallmark of senescent T cells [74]. Similar studies have shown that changes in T lymphocyte metabolism and inflammation accumulation can lead to premature senescence and the development of various “senescence diseases” in animals. Regulating immune cell metabolism or reducing inflammation may slow senescence and treat age-related diseases [75]. Reduced autophagy is central to immune senescence, with decreased autophagy impairing memory B-cell responses in older individuals. In the elderly, decreased spermidine (SPD) levels result in reduced cell autophagy and expression of key transcription factors. Endogenous factors can compensate for reduced SPD by promoting eIF5A and TFEB translation, inducing autophagy, and restoring memory B cells in older mice and humans to a youthful state [76]. These studies show that during body senescence, T and B lymphocytes, and the innate immune system, undergo senescence and immune disorders, which accelerate aging and increase disease risk, including pathogen invasion, tumor development, and autoimmune diseases, significantly affecting quality of life and longevity.

## 4. The Causes of Cancer Cellular Senescence

Cancer is characterized by genomic instability in somatic cells, including variations such as gene mutations, chromosome rearrangements, and gene copy number variations, which drive cancer development. It is well known that cancer cells exhibit a “continuous proliferation signal” [12]. While cancer cells are often thought to have infinite growth potential, they can, in some cases, exhibit age-related characteristics. These characteristics include OIS and drug-induced senescence, which can be triggered by chemotherapeutic agents, epigenetic modulators, telomerase inhibitors, and cell cycle inhibitors (Table 1).

### 4.1. Oncogene Gene-Induced Senescence

In 1997, researchers first demonstrated that oncogene activation (HRAS^V12^) induces growth arrest, known as OIS, by enhancing cyclin-dependent kinase inhibitors such as p16^Ink4a^ and p21 [7]. These inhibitors form a natural barrier to cancer development and play a role in its prevention. Additionally, carcinogenic Ras-induced histone H3 lysine 9 methylation (H3K9me) of heterochromatin leads to stunted growth and promotes cellular senescence. The H3K9me-mediated senescence associated with cancer genes includes retinoblastoma (Rb) as an early developmental barrier [77]. Moreover, the activation of the B-Raf proto-oncogene (BRAF) due to mutation by the oncogene v-RAF leads to increased p16^Ink4a^ expression, positive senescence-associated beta-galactosidase staining, and stagnation of melanoma cell growth [78]. Recent studies have found that inactivation of the MYC oncogene accelerates premature senescence in mice and reduces cancer incidence [79]. OIS can occur independently of the p53 and DNA damage signal pathways, including INK4A-RB [80]. Inactivation of the tumor suppressor gene phosphatase and tensin homolog (PTEN) induces growth arrest both in vitro and in vivo through the p53-dependent senescence pathway, leading to senescence in primary prostate epithelial cells, a process known as PTEN loss-induced cellular senescence [81]. This indicates that PTEN loss inhibits carcinogenesis by inducing p53 expression, a crucial fail-safe protein in PTEN-deficient tumors.

### 4.2. Drug-Induced Senescence

Cancer cells have the ability to proliferate indefinitely and evade senescence. However, clinical approaches have shown that specific conventional anticancer therapies can induce a senescence-like state in these transformed cancer cells, known as TIS, both in vitro and in vivo [82]. TIS could potentially be an effective treatment for both early and advanced stages of cancer. Among these therapies, conventional anticancer treatments like chemotherapy and radiotherapy can induce senescence in cancer cells. However, while high-dose chemotherapy can eradicate most cancer cells, it can also cause significant damage to surrounding stromal cells, including immune and vascular endothelial cells, which may lead to increased cancer recurrence and metastasis. “Non-lethal-dose chemotherapy” is a newer approach that involves using lower doses of chemotherapy to reduce toxic side effects, improving patient tolerance and potentially enhancing efficacy. Recent studies show that cells exposed to non-lethal doses of chemotherapy undergo significant changes in morphology and metabolic activity, leading to a tendency to enter a senescent state [83]. During cancer treatment, low doses of chemotherapy can trigger a senescent state in cancer cells, while high doses typically induce apoptosis [84,85]. The induction of apoptosis or senescence depends partially on the level of stress imposed on cancer cells. Lower levels of damage may lead to senescence-related antiproliferative responses without triggering apoptosis. For instance, in prostate cancer cell lines, high doses of doxorubicin induce apoptosis, while lower doses induce TIS [86,87]. Molecularly, most cases of TIS are caused by DNA damage induced by drugs in cancer cells. This damage is mediated by the activities of ATM, ATR, CHK1, and CHK2, as well as the CDKN2A/p16^Ink4a^/p19ARF tumor suppressor [88]. Certain chemotherapeutic drugs, including doxorubicin, etoposide, and camptothecin, cause extensive DNA damage and increase the expression of p53 and its downstream target CDKN1A in cancer cells, leading to senescence [89,90]. Methotrexate and gemcitabine also induce genotoxic stress by inhibiting DNA synthesis, thereby causing cellular senescence in cancer cells [91,92]. The latest report indicates that the standard treatment for prostate cancer (PCa) using Docetaxel can induce robust senescence responses. Besides chemotherapy, radiotherapy is also a common treatment for various cancer types. Radiotherapy can cause irreparable DNA damage responses, activating both apoptosis and cellular senescence through the ATM and p53-p21 pathways [93]. These studies suggest that both chemotherapy and radiation treatments induce cancer cell senescence primarily through DNA damage responses.

Cancer cells frequently exhibit elevated levels of cyclin-dependent kinases (CDKs) to sustain their continuous proliferation ability [94]. In contrast, senescent cells exhibit increased levels of CDK inhibitors, such as INK4A and p21, which leads to cell cycle arrest. Consequently, protein drugs are often utilized in cancer treatments that induce senescence to inhibit CDKs or enhance CDK inhibitor levels. For instance, under normal conditions, cyclin D and CDK4/6 form active kinase complexes that phosphorylate Rb protein to regulate the transition from the G1 phase to the S phase. However, the use of CDK4/6 inhibitors (CDK4/6i) can result in cell overgrowth, activation of p53 due to osmotic and replication stress, and an increase in its downstream target, p21, which promotes permanent cell cycle exit and induces senescence in cancer cells [95]. CDK2 is another cyclin-dependent kinase that primarily functions during the G1 and S phases of the cell cycle. Similar to CDK4/6, CDK2 directly phosphorylates Rb and other substrates essential for the G1-to-S-phase transition, DNA replication initiation, DNA repair, and exit from the S phase. In cancer treatments, CDK2 inhibitors can specifically target CDK2 in cancers where it is a primary driver, minimizing the toxicity and dose limitations associated with inhibiting CDK4 and CDK6. Research has shown that the selective CDK2 inhibitor INX-315 can induce cell cycle arrest and senescence in solid tumors [96].

As noted earlier, telomere shortening and epigenetic abnormalities contribute to normal cell senescence. Therefore, inhibiting telomerase or modifying the epigenome to induce cancer cell senescence may be effective strategies. To delay cellular aging, normal cells use the enzyme telomerase to replenish lost telomeres and maintain their length. Consequently, a deficiency in telomerase leads to cellular senescence. In contrast, cancer cells exhibit high telomerase activity, which allows them to continue dividing and evade senescence. Cancer cells often reactivate telomerase to evade replication senescence [97]. Several compounds that inhibit telomerase complexes have been identified as potential candidates for anticancer therapy [98]. One such compound is 6-thio-2′-deoxyguanosine (THIO), a nucleoside analog that is readily incorporated into newly synthesized telomeres by telomerase. THIO has demonstrated antitumor activity in gliomas [99]. Furthermore, modulating the epigenome of cancer cells can also induce senescence. DNA methylation in cancer cells is complex and specific to gene expression regulation; specific DNA methylation patterns contribute to tumor progression and histone modifications [100]. Thus, targeting DNA methylation to regulate senescence gene expression in cancer cells could be a viable strategy for suppressing cancer development. Inhibiting DNA methyltransferases can lead to increased methylation in the CpG-rich promoter regions of cyclin-dependent kinase inhibitor A (CDKN2A), inducing the expression of INK4A and ARF, which results in cancer cell senescence [101]. Recent research has also identified that lncRNA lncPEAT in gliomas interacts with the ubiquitin-digesting enzyme USP16, suppressing USP16′s recruitment to chromatin and thereby inhibiting the expression of senescence-related genes CDKN1A and CLUSTERIN [102]. This suggests that developing small molecule inhibitors targeting lncPEAT, which can inhibit its overexpression or promote senescence in glioblastoma (GBM) cells, may offer therapeutic benefits for glioma. These findings indicate that targeting epigenetic modifications, such as DNA methylation and histone modifications, to regulate cancer cell senescence is a promising strategy. Further research into the mechanisms of epigenetic modifications in regulating senescence and cancer is crucial for developing specific drug targets.

## 5. The Paradoxical Role of Cellular Senescence in Cancer

**Figure 2:**
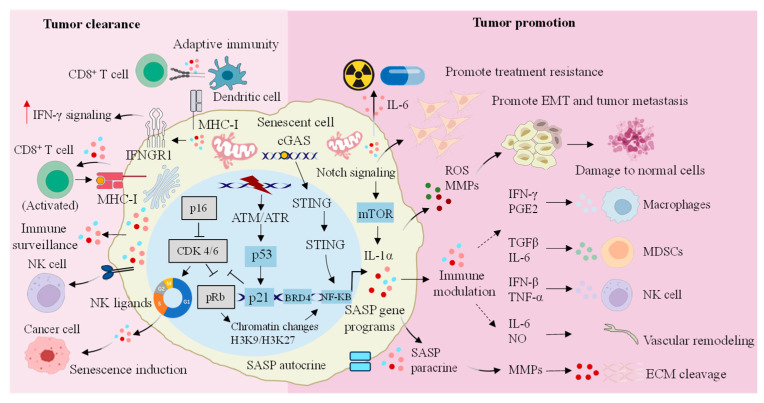
Dual regulatory role of senescent cells in cancer progression. SASP secreted by senescent cells not only plays an anticancer function by enhancing the activity of a series of immune cells, but also promotes the occurrence of cancer by promoting the immune escape and drug resistance of cancer cells. IFN-γ: interferon-γ; IFNGR1: IFN-gamma receptor 1; MHC: major histocompatibility complex; NK: natural killer; cGAS: cyclic GMP-AMP synthase; STING: stimulator of interferon genes; CDK: cyclin-dependent kinase; pRb: retinoblastoma protein; ATM: ataxia telangiectasia mutated; ATR: ataxia telangiectasia mutated and Rad3-related; NF-κB: nuclear factor κB; BRD4: bromodomain-containing protein 4; SASP: senescence-associated secretory phenotype; H3K9/H3K27: histone H3 lysine 9/histone H3 lysine 27; mTOR: mechanistic target of rapamycin; IL-1α: interleukin-1α; EMT: epithelial-to-mesenchymal transition; ROS: reactive oxygen species; MMPs: matrix metalloproteinases; PGE2: prostaglandin E2; TGF-β: transforming growth factor-β; IL-6: interleukin-6; IFN-β: interferon-β; TNF-α: tumor necrosis factor-α; NO: nitric oxide; MDSCs: myeloid-derived suppressor cells; ECM: extracellular matrix.

Senescence is associated with various diseases, including neurodegenerative conditions such as Alzheimer’s disease (AD) and Parkinson’s disease, cardiovascular diseases, and many types of cancer [103]. In these conditions, cancer and senescence share several common features, including genome instability, epigenetic changes, chronic inflammation, and ecological imbalance. These overlapping characteristics and markers are often referred to as “meta-features” [10]. The incidence of cancer is relatively low in individuals aged 0–34, rises significantly in the 35–39 age group, and peaks in those aged 80–84. This trend indicates a strong association between cancer occurrence and aging [104]. Elderly individuals with cancer often have complications and a higher incidence of age-related diseases compared to those without cancer [105]. Certain genetic syndromes reveal a shared genetic basis for senescence and cancer, suggesting that premature senescence can be associated with the development of various malignancies [106]. These findings imply that the relationship between senescence and cancer development is bidirectional. A thorough understanding of the interplay between cellular senescence and cancer cells is essential for effective cancer treatment and prevention (Figure 2).

### 5.1. Senescent Cells Suppress Tumors

While senescent cells exhibit proliferation stagnation, cancer cells are characterized by their capacity for unlimited growth. Research shows that while tumor cells and senescent cells share some common features, such as genomic instability, epigenetic changes, and chronic inflammation, they also exhibit some opposing characteristics. Senescent tumor cells continue to have active metabolism and release SASP factors, including various cytokines and inflammatory molecules, into the tumor microenvironment. The SASP factors secreted by tumor cells can stimulate an immune response, creating a pro-inflammatory environment that attracts immune cells, facilitates the immune clearance of the tumor cells, and aids in the removal of abnormal cells to restore tissue homeostasis. Therefore, when tumor cells exhibit senescence-related traits, such as telomere shortening or stem cell dysfunction, they may experience cell cycle arrest and reduced tumor formation by triggering an immune response via SASP.

#### 5.1.1. Growth Retardation

OIS is a senescence process driven by the activation of oncogenes. OIS results in a stable growth arrest of premalignant cells, reinforced by cyclin-dependent kinase inhibitors like p16^Ink4a^ and p21, thereby acting as a natural barrier to tumor development. Senescent cells, including those undergoing OIS, inhibit tumor growth through metabolic changes and the SASP [13]. Cyclin-dependent kinase 7 (CDK7) is a catalytic subunit of the CDK-activating kinase (CAK) complex, which activates CDK1, 2, 4, and 6 to support the cell cycle. Genome-wide screening using CRISPR has identified that CDK7 inhibitors, such as samuraciclib, are key determinants in inducing cancer cell senescence, particularly through the mTOR signaling pathway. Increased cell size may drive cell cycle arrest and induce permanent cell cycle exit. Increased growth signaling makes cancer cells, but not normal healthy cells, more sensitive to CDK7 inhibitors [107]. The p53/p21^CIP1^ and p16^Ink4a^/Rb tumor suppressor pathways are fundamental to the growth arrest associated with cellular senescence. Research has shown that the senescence regulator XPO7 affects senescence by modulating p21^CIP1^ induction. Deletion or mutation of XPO7 delays cancer cell senescence, accelerating liver cancer progression [108]. This suggests that some genes involved in cellular senescence might also function as tumor suppressors during cancer development. Furthermore, replication senescence disrupts cell proliferation, as the absence of cell cycle checkpoints prevents the activation of senescence, leading to continuous proliferation, telomere shortening, and genome instability. Recent research has found that telomeres play a role in mediating tumor suppressor functions during cellular senescence. A dysfunctional telomere replication crisis activates two intertwined cytoplasmic DNA sensing mechanisms and triggers an interferon (IFN) response. Disruption of fused telomeres and subsequent release of nuclear DNA into the cytoplasm initiate the cGAS-STING pathway and the expression of interferon-stimulated genes (ISGs), leading to cell death [109]. This suggests that during cellular senescence, dysfunctional telomeres activate nucleic acid sensors and induce cell death to suppress cancer cell growth.

#### 5.1.2. Immune Surveillance

The immune system recognizes, kills, and eliminates mutant, senescent, and cancer cells in the body in time, namely immune surveillance. Studies have shown that mice with immune surveillance disorders tend to accumulate more senescent cells and develop chronic inflammation compared to normal mice. These mice also showed a higher incidence of age-related diseases and a lower survival rate than normal mice [110]. This suggests that impaired immune surveillance may accelerate cellular senescence and potentially lead to cancer. SASP factors, including chemokines, cytokines, growth factors, and enzymes, can induce paracrine senescence or stable proliferation arrest in adjacent cancer cells, and some of these factors can enhance immune surveillance. For example, the secretion of the inflammatory cytokine interleukin-1α (IL-1α) by senescent cells induced by oncogenes and therapy can trigger an autocrine inflammatory response by activating NF-κB, leading to the transcription of interleukin-6 (IL-6) and interleukin-8 (IL-8), which further promotes immune activation [111]. Additionally, IL-1α mediates paracrine senescence in adjacent cells, helping to inhibit tumor progression [112]. Furthermore, IL-1α, IL-6, and IL-8 mediate the recruitment of M1-like macrophages, helper T cells, and NK cells into the TME, further driving the clearance of cancer cells [113]. Cell senescence can induce senescence in primary cancer cells, enhancing their ability to activate autologous antigen-specific tumor-infiltrating CD8(+) lymphocytes. For example, cellular immunogenicity associated with senescence is increased. In mice with KRAS^G12V^-induced senescence, hepatocyte proliferation was halted. This condition promotes the recruitment of macrophages and T cells, which are subsequently cleared by cytotoxic T lymphocytes (CTLs) [114]. Senescent cells can also effectively activate DCs and antigen-specific CD8(+) T cells. Recent studies indicate that p53-mediated senescence increases the number of macrophages and lymphocytes in a mouse model of p53 inhibition and liver cancer recovery, suggesting a shift from a “cold” to a “hot” tumor microenvironment. Additionally, senescent cells can increase the expression of MHC-I molecules and activate interferon-γ (IFN-γ) signaling, efficiently activating DCs and antigen-specific CD8+ T cells to promote tumor regression [115]. This demonstrates that senescent cells can positively influence anticancer immunotherapy. Similarly, in a senescence-activated CD8(+) T-cell-mediated liver cancer model, researchers found that senescence can alter the cell surface proteome, affecting how tumor cells respond to environmental factors. Compared to proliferative cells, senescent cells can more strongly upregulate IFN-γ receptors and enhance antigen presentation mechanisms. Conversely, disrupting IFN-γ induction in senescent cells reduces their immune-mediated clearance ability without affecting the senescence state or its characteristic secretory processes [116]. Moreover, senescent cells can use SASP to recruit immune cells and promote immune clearance. Tumor cells are often exposed to stressors like hypoxia and ROS that can induce senescence. Cytokines released by immune cells can also trigger senescence in tumor cells. Senescent cancer cells, due to their reduced or absent proliferation ability, do not drive tumor growth directly but alter the TME through their SASP. Notably, the SASP from tumor cells can recruit and activate CD4(+) and CD8(+) T cells, providing anti-tumor protection [117,118]. In summary, these studies suggest that senescent cells can boost immune surveillance by activating immune responses, increasing cancer cells’ sensitivity to IFN-γ, and improving the TME, which can lead to effective tumor regression.

### 5.2. Senescence and Tumor Promotion

For thousands of years, humans have been pursuing the perfect unity of their own health and longevity. Despite efforts to understand senescence at the cellular and molecular levels and to find ways to delay or reverse it, senescence remains an irreversible process. Unfortunately, senescence is irreversible, and its decline in biological function is associated with a higher risk of age-related diseases such as cancer, diabetes, and hypertension. Biologically, senescence is the inevitable outcome of ongoing damage accumulation at the molecular and cellular levels over time. Conversely, tissue- and organ-level retrogressive changes lead to a gradual decline in physical fitness and cognitive abilities, with an increasing individual cancer risk towards the end of life [119]. In cancer patients, higher levels of senescence-related proteins correlate with lower survival rates, suggesting a close link between senescent cells and cancer cell growth [120]. This implies a risk connection between senescence and cancer, with increasing age correlating with a higher cancer risk. To explore the relationship between senescence-related SASP and cancer, comparisons between senescent and control cells revealed differences in the distribution of two genes, *METTL3* and *METTL14*, which are closely related to SASP secretion. In normal cells, *METTL3* and *METTL14* are located far apart, but in senescent cells, they are closer to each other and near the promoter regions of SASP-related regulatory genes. This proximity creates a strong promoter loop, enhancing the expression of SASP-related regulatory genes. This, in turn, promotes cancer development [121]. This strongly suggests that senescence-related SASP is closely associated with cancer development. Molecularly, the accumulation of senescent cells in the TME can drive tumorigenesis through paracrine signaling mediated by SASP [122]. Certain SASP factors can alter epithelial–mesenchymal transformation and migration of adjacent tumor cells, as well as enhance tumor resistance to immunotherapy through potent immunosuppressive mechanisms, leading to immune escape post-treatment [123].

#### 5.2.1. Senescence-Mediated Remodeling of Tumor Microenvironment

The TME, which includes fibroblasts, endothelial cells, pericytes, adipocytes, extracellular matrix (ECM), and immune cells, plays a crucial role in cancer development. Fibroblasts are predominant matrix components in the body. SASP factors released by senescent cells, including cytokines, chemokines, growth factors, and proteases, can reprogram primary and metastatic microenvironments, enhancing cancer cell invasion and metastasis [124]. Studies have shown that senescent fibroblasts are more prevalent in individuals over 60 compared to those under 40. An increase in senescent fibroblasts reduces cytokeratin and E-cadherin expression, facilitating epithelial–mesenchymal transition [125]. SASP-related fibroblasts experience major metabolic shifts, including mitochondrial dysfunction, hydrogen peroxide production, and a transition to aerobic glycolysis [126]. These metabolic changes lead to increased lactic acid production, boosting cancer cell invasiveness and accelerating age-related damage [127], which supports a cancer-friendly metabolic environment. In tumor development, many senescent fibroblasts can convert into cancer-associated fibroblasts (CAFs), which drive malignant growth, invasion, and metastasis. Senescent cells participate in ECM remodeling by disrupting ECM secretion, degrading it through enzymes, and impairing elastin and collagen networks, which reduces tissue tension and elasticity, thus fostering a tumor-friendly microenvironment. Cancer cells can navigate along ECM scaffolds altered by senescence [128]. On the flip side, senescence-induced microenvironment changes can prompt inflammatory cells to activate resident fibroblasts, resulting in excessive ECM secretion and cross-linking, which enhances matrix stiffness. This increased stiffness creates a physical barrier that hinders immune cell infiltration and alters vascular function, accelerating tumor growth and immune evasion [129]. SASP factors like IL-6 and IL-8 are key in mediating the tumor-promoting effects of senescent cells, fostering a chronic inflammatory microenvironment that aids cancer growth. These factors also promote the expression of matrix metalloproteinases (MMPs), which drive epithelial-to-mesenchymal transition and increase tumor invasiveness [130,131].

Interestingly, during clinical treatment, several senescent cells in the TME emerged as major sources of persistent inflammatory factors. One such factor is serine protease inhibitor Kazal type I (SPINK1), a soluble protein highly synthesized and released by stromal cells, which enters the damaged microenvironment, alters the phenotype of residual cancer cells, and promotes drug resistance, leading to resistance to clinical treatment. Additionally, fluctuations in SPINK1 levels in peripheral blood are closely linked to patient survival [132]. This indicates that senescent cells in the TME affect stromal cells, interfering with cancer cell growth and contributing to therapeutic resistance. Recent studies using a transgenic mouse model with the cell senescence marker gene (Ink4a/Arf) have identified senescent cells in GBM in both patients and mice [133]. Similarly, using a brain-like organ and CDKN2A-DTR mouse model showed that radiotherapy-induced DNA damage in normal astrocytes around GBM led to cell senescence, marked by increased expression of p16^Ink4a^, p21^CIP1^, and other cell cycle inhibitors. These cells also released substantial SASP, activating key signaling pathways in GBM cells, which mediated the secretion of chemokines to recruit myeloid cells and create an immunosuppressive microenvironment. This process reshapes the TME and contributes to tumor recurrence [134]. These studies demonstrate that TME remodeling by senescence-related SASP is a significant factor in cancer cell invasion and metastasis.

#### 5.2.2. Immune Escape Mediated by Cell Senescence

Senescent cells undergo numerous metabolic reprogramming events that alter their intracellular environment. In breast cancer, senescence results in abnormal alterations in intracellular pH (pHi) and lysosomal pH (pHL). An increase in pHL then impairs lysosomal function and reduces lysosome quantity, which impacts the expression of immune-related genes [135]. This suggests that disruptions in homeostasis in senescent cells may influence the expression of immune-related genes. Activation of CD8(+) T cells as effector cells and co-culturing with mouse primary lung fibroblasts (MPFs) revealed heterogeneous PD-L1 expression in senescent MPFs. Post co-culture, senescent cells exhibited greater sensitivity to T cells compared to resting cells. Transcriptome sequencing (RNA-seq) was employed to identify transcriptional differences between senescent and resting MPFs. The results showed significant enrichment of pathways related to inflammation, cytokine production, T-cell activation, and antigen presentation in senescent MPFs. Senescent cells can stimulate cytotoxic T-cell immunity through the secretion of pro-inflammatory cytokines and presentation of internal antigens, and PD-L1 expression in senescent cells is crucial for evading T-cell immunity. Furthermore, weakening the inhibitory signals on T cells could reactivate immune surveillance and lead to the elimination of senescent cells, potentially improving various age-related phenotypes [136]. Moreover, prolonged senescence and chronic SASP induction can result in a similar immune escape mechanism. For instance, the BRAFV600E series mutant melanocytic nevus ages while evading immune surveillance, leading to its accumulation with age in humans [78]. These studies imply that senescent cells might facilitate the immune escape of cancer cells due to diverse changes in metabolism, structure, and homeostasis. Failure of the tumor immune response to eliminate senescent cells may worsen cancer progression.

Immune senescence, characterized by thymus atrophy and alterations in T-cell quantity and quality, is a significant factor contributing to various age-related diseases, including cancer. Age-related immune senescence primarily affects effector T cells and other immune cells crucial for tumor immunity. These changes can result in the activation and infiltration of additional immunosuppressive cells in older individuals, potentially increasing their susceptibility to cancer and metastasis. The TME harbors immune cells, and immune cell senescence impacts their function, contributing to the immune escape of cancer cells. Researchers investigated the relationship between senescence and immune cell aging by knocking out a critical gene in mouse immune cells. This gene knockout resulted in mouse immune cells exhibiting age-related senescence characteristics. Essentially, the mouse immune cells exhibited signs of “premature senescence.” Beyond immune organs, DNA damage and senescence were observed in other organs of the mice, leading to a shorter lifespan [64]. This suggests that age-related immune senescence primarily affects effector T cells and other immune cells crucial for tumor immunity. These alterations may result in the activation and infiltration of additional immunosuppressive cells in older individuals, potentially heightening their susceptibility to cancer and metastasis. NK and natural killer T (NKT) cells are crucial subsets of the immune system. In mouse models of E0771 breast cancer and B16 melanoma, researchers found that NK and NKT cells play dynamic roles in the immune response to cancer. During the early stages of tumor development, NK and NKT cells exhibit effective characteristics. In later stages of cancer, NK cells become senescent and NKT cells (excluding iNKT) are depleted, leading to impaired cytotoxicity and dysfunction of NK and NKT cells, which promotes the immune escape of cancer cells [137]. This suggests that NK cells suffer impairment during senescence. The same study identified retinoic acid receptors (RARs) as nuclear receptors that regulate gene expression in response to retinoic acid, a metabolite of vitamin A. RAR activation has demonstrated anti-tumor effects in various tumor models. Using a retinoic acid receptor agonist can boost the effect of cell senescence in prostate cancer cells and work in conjunction with docetaxel to inhibit tumor senescence and reprogram the senescence-related secretory phenotype, thereby activating the anti-tumor immune response and enhancing NK cell anti-tumor effects [138]. Like NK cells, T-cell senescence impacts their tumor-killing effectiveness. In aged mice, increased ceramide accumulation in T-cell mitochondria leads to T-cell dysfunction. These T cells lose their strong killing ability in animal models, significantly weakening their capacity to kill tumor cells. Blocking ceramide secretion and reducing its content in T cells allowed these senescent T cells to regain their killing ability, potentially achieving an anti-tumor effect similar to that of young T cells [139].

Additionally, macrophages exhibiting senescence with tumor-promoting activity have been identified in KRAS-driven lung cancer mouse models. These macrophages displayed traits of cell senescence and SASP in normal senescent lungs, and macrophages with senescence markers were also observed in human precancerous lung tumors. Macrophages and endothelial cells are the primary types of senescent cells in KRAS-driven lung tumors in mice. Research has shown that removing senescent cells or macrophages can significantly decrease tumor burden and enhance survival rates [140]. Significantly, during tumor development, a subset of key senescent macrophages with high C-X-C chemokine receptor type 1 (CXCR1) expression has been identified. These macrophages, characterized by high CXCR1 expression, can suppress cytotoxic T-cell proliferation and facilitate tumor progression. Targeted elimination of these senescent macrophages can impede tumor progression [141]. These studies demonstrate that immune cell senescence can cause functional impairment and contribute to cancer cell immune escape.

## 6. Senescence Therapy of Tumor Cells

As previously mentioned, cell senescence results in growth arrest and is associated with an immune response activated by SASP. Thus, in the early stages of cancer progression, inducing senescence in cancer cells appears to be an effective strategy to inhibit tumor growth. Persistent DNA damage from conventional chemotherapy, radiotherapy, CDC7 inhibitors (such as XL413 or TAK931), or telomerase inhibitors (like GRN163L or BIBR15) typically results in the induction of senescence [142,143]. Additionally, extensive DNA damage responses can activate ATM or ATR signaling pathways, leading to the activation of p53 and p21, and thereby inducing cancer cell senescence [144]. Furthermore, drugs such as the PTEN inhibitor VO-OHpic (VO), Mdm2-p53 interaction inhibitor nutlin 3, RG7112, and UBX0101 can induce cancer cell senescence via the p53-p21 signaling pathway [145]. Early studies have shown that combining the MEK inhibitor trametinib with the CDK4/6 inhibitor palbociclib (T/P) enhances anti-tumor effects by inducing tumor cell senescence through Rb and activating NK cells [146]. The T/P combination can induce senescence in pancreatic ductal adenocarcinoma (PDAC) associated with *KRAS* gene mutations. This combined therapy can prompt PDAC tumor cells to secrete various SASP factors, including angiogenic factors (VEGF), platelet-derived growth factor (PDGFA/B), fibroblast growth factor 2 (FGF2), and matrix metalloproteinases (MMP-2/3/7/9/10), which subsequently enhance endothelial cell proliferation, tubular structure formation, and vascular remodeling [147]. This suggests that inducing senescence in cancer cells could facilitate vascular remodeling and inhibit tumor progression. Likewise, docetaxel, a standard treatment for prostate cancer, elicits a robust senescence response. Recent research indicates that RAR agonists can amplify the senescence induced by docetaxel in prostate tumor cells and reprogram SASP from promoting tumor growth to inhibiting it [138]. This implies that combining RAR agonists with docetaxel can enhance intratumoral NK cell recruitment and promote tumor clearance by NK cells, highlighting the potential of senescence-inducing therapies in cancer treatment.

However, dysfunctional senescent cells not only occupy normal tissue space but also release factors that can damage the body and contribute to cancer development. This suggests that the persistence of treatment-induced senescence in cells may be detrimental. Therefore, in advanced cancer stages, minimizing tumor progression and avoiding adverse reactions can be achieved by removing treatment-induced senescent cells or excessive senescence in cancer cells [148]. Senolytic therapies, developed through drug research, aim to remove senescent cells, collectively known as senolytics [149]. A characteristic of senescent cells is altered chromatin structure, leading to changes in gene expression. These changes can impact fundamental processes like apoptosis regulation, creating new vulnerabilities in senescent cells that can be targeted by anti-senescence agents to prevent cancer cell senescence accumulation [150]. Significantly, treatment-induced senescence can impact cancer cells and surrounding tissues by causing inflammation, recruiting immune cells, spreading senescence to other cells, and altering immune cell functions both positively and negatively. Moreover, SASP can differ among senescent cancer cells based on tissue type, the method of senescence induction over time, and hormonal responses, which may influence the effectiveness of immunotherapy. Thus, cell senescence and immunotherapy are interrelated and jointly influence cancer development. Recently, cancer therapy involving NK cells has rapidly expanded and become a major area of innovation in immunotherapy. NK cells are among the first immune cells to respond to tumors. Studies have shown that NK cells can extend lifespan by 20% to 30% by eliminating senescent cells in animals. In comparison to animals that retained senescent cells, those cleared of them were approximately six months younger, which corresponds to decades in human terms. Expanded and cultured autologous NK cells demonstrated a strong ability to kill tumor cells and remove senescent cells, and also reduced PBMC senescence markers in the blood after reinfusion [151]. Following NK cell autotransfusion in healthy adults (ages 36–71), the proportion of senescent CD3+ T cells and several inflammatory markers showed significant improvement [152]. These findings suggest that combining senescence-inducing therapies with immunotherapy could potentially enhance cancer treatment outcomes.

## 7. Discussion and Prospect

Cancer cells gain the ability to proliferate uncontrollably due to various carcinogenic signals. Mutations in key genes or genomic instability due to epigenetic changes drive cell carcinogenesis and ongoing proliferation, while metabolic reprogramming allows cancer cells to obtain energy from external sources. Interactions between carcinogenic signaling pathways and metabolic reprogramming can lead to TME remodeling and disrupt immune cell functions, promoting immune escape [153]. Thus, from the perspective of cancer cell development, excessive DNA damage or inadequate repair due to internal and external factors leads to gene mutations that affect protein homeostasis through transcription and translation. These mutations alter intracellular signaling pathways, metabolic pathways, and immune activation by disrupting biological functions, resulting in complex signaling networks with both positive and negative feedback mechanisms in cancer cells. Notably, there are similarities and antagonistic features between cancer characteristics and those of cell senescence. As a result, research into treating cancer by inducing cell senescence is gaining popularity. However, in clinical practice, SASP production is a key feature of both normal and cancer cells post-senescence, with SASP exerting dual regulatory effects on the TME, immune cells, and tissue homeostasis. The impact of SASP on cancer cells depends significantly on the environment and cell type, and varies at different stages of cancer progression [154]. In the early stages of carcinogenesis, SASP generally inhibits tumors, while in advanced stages, SASP tends to promote tumor growth [113]. Therefore, treatments designed to induce cancer cell senescence need to account for the varying degrees and stages of cancer progression.

Over time, SASP in senescent cancer cells is predominantly harmful, promoting tumor growth, drug resistance, immunosuppression, metastasis, and angiogenesis. Senescent cancer cells can remain dormant for extended periods, avoiding treatment and increasing the risk of tumor recurrence [64]. Genotoxic chemotherapies can induce senescence in both cancer and normal cells, leading to severe side effects. Persistent senescent normal cells can cause local and systemic inflammation through SASP, exacerbating the side effects of chemotherapy [155]. Notably, immune cells in the TME exhibit senescence and functional exhaustion, often referred to as a “cold” TME. This “cold” TME disrupts the secretion and function of immune cells and factors. The status of specific tumor suppressor genes, especially p53, in cancer cells can greatly influence how senescence-induced inflammation affects tumor growth, either inhibiting or promoting it. These genes can also interfere with metabolic pathways by interacting with metabolic enzymes, impacting the TME and influencing the senescence microenvironment [156]. Thus, combining anti-senescence therapies with other treatments may be beneficial in cancer management. Besides directly targeting cancer cells and mitigating chemotherapy side effects on normal tissues, developing senolytics to remove early pre-tumor senescent cells or other senescent cells in the TME could help counteract the harmful effects of SASP.

## Figures and Tables

**Table 1:** The causes of senescence in normal cells and cancer cells.

Normal Cells		Cancer Cells	
**Genome Stability**		**Chemotherapy and radiation therapy induction**	
** 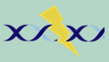 **	Exogenous (chemical, physical, biological agents) Endogenous (DNA replication errors, chromosomal segregation defects)	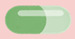	Doxorubicin, etoposide, et al. Inducing DNA damage Blocking DNA synthesis Cyclin dependent kinase (CDK) inhibitors CD4/6 inhibitor CDK2 inhibitor Telomere targeting inhibitors Telomerase substrate precursor nucleoside analogue Epigenetic regulation DNA methyltransferase inhibitor
**Telomere depletion**		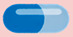	
** 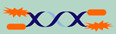 **	Cell replication (telomere loss) Telomere dysfunction (DNA damage)	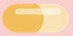	
		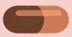	
**Epigenetic changes**		**Oncogenic signaling**	
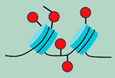	DNA methylation patterns, Post-translational modifications Chromatin remodeling non coding RNA (ncRNA)	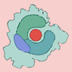	RAS, MYC, BRAF and PTEN, et al induced cancer cell senescence
**Metabolic changes**		**Immune cytokine induction**	
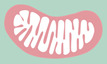	Glucose metabolism Amino acid metabolism Fatty acid metabolism ATP, NAD+	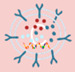	Immune factors produced by immune cells induce cancer cell senescence (IFN-γ, TNF)
**Signaling Pathway**		**Hypoxia and reactive oxygen species induction**	
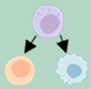	PARP1/AMPK pathway ROS/AMPK/mTOR pathway SIRTs mediated signaling pathways	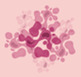	Hypoxic conditions can induce upregulation of HIF-1 α, thereby inducing cancer cell senescence
